# Chronic pain in Gaucher disease: skeletal or neuropathic origin?

**DOI:** 10.1186/s13023-017-0700-7

**Published:** 2017-08-31

**Authors:** Grazia Devigili, Michele De Filippo, Giovanni Ciana, Andrea Dardis, Christian Lettieri, Sara Rinaldo, Daniela Macor, Alessandro Moro, Roberto Eleopra, Bruno Bembi

**Affiliations:** 1 Department of Neurology, AMC Hospital of Udine, Piazzale Santa Maria della Misericordia 15, 33100 Udine, Italy; 2 Regional Coordinator Centre for Rare Diseases, AMC Hospital of Udine, Building 16; Piazzale Santa Maria della Misericordia 15, 33100 Udine, Italy

**Keywords:** Gaucher type 1 disease, Bone pain, Neuropathic pain, Small fibre neuropathy, Peripheral neuropathy

## Abstract

**Backgound:**

Pain is one of the most disabling symptoms of Gaucher disease. It is referred by the majority of Gaucher patients and often persists despite long-term enzyme replacement treatment. It has been mainly considered as nociceptive pain secondary to skeletal involvement but it is described even in the absence of bone disease without a clear explanation. In the last years an increasing number of reports have described the presence of neurological manifestation in Gaucher type 1 patients, including subclinical large fibre neuropathy. In our Gaucher clinic we have observed the recurrence of painful symptoms in a group of type 1 Gaucher patients even after a long-term enzyme replacement therapy.

**Methods:**

A cross-sectional study was designed to investigate the pathophysiology of pain in a cohort of 25 Gaucher patients (13 females, 12 males). Twenty-two patients received enzyme replacement therapy for a period of time ranging from 10 to >20 years, while three were new diagnosis. Pain was classified as bone or neurologic related on the basis of anamnestic data, clinical and electrophysilogical examinations. Intensity and quality of pain were recorded by Douleur Neuropathique en 4 questionnaire and Neuropathic Pain Symptom Inventory. Neuroalgological evaluation, quantitative sensory testing, nerve conduction studies and evaluation of epidermal nerve fibres density were performed. Comorbidities for peripheral neuropathy were excluded.

**Results:**

Thirteen patients complained of pain suggestive of neuropathic origin with proximal patchy distribution, six manifested severe pain paroxysmal, nine pinprick hypoesthesia and 17 thermal hypoesthesia. At quantitative sensory testing, all of them showed high cold thresholds with *errata sensation* (burning instead of cold), paradoxical heat sensation and mechanic hypoesthesia; three patients showed pressure pain hyperalgesia. Epidermal denervation was present in 19 patients, 12 of them with non-length dependent pattern.

**Conclusions:**

These results confirm the role of peripheral neuropathy in Gaucher pain and demonstrate that skin denervation is as a constitutive feature of the disorder. In addition, they further confirm the existence of a *continuum Gaucher phenotype,* and provide a new interpretation of pain origin that should be considered for an appropriate disease management and to avoid unnecessary dose escalations of enzyme therapy.

## Background

Gaucher disease is a lysosomal storage disorder due to the deficient activity of the acid β-glucosidase enzyme [1]. Based on the presence of neurological involvement, three phenotypes have been described: the non-neuropathic form (GD1), the acute, early fatal, neuropathic form (GD2) and the chronic late-onset neurological form (GD3) [1, 2]. Although this classification helps in describing disease progression and prognosis, studies of GD clinical history showed the existence of a *continuum* of phenotypes, ranging from the severe GD2 to the asymptomatic GD1 form [3]. In accordance with this concept, different authors have described signs of neurological involvement in GD1 patients [4–9]. In particular, Biegstraaten M et al. showed a high prevalence of peripheral neuropathy (16.5%) in a cohort of GD1 patients followed for 2 years [9].

A relevant manifestation of GD is pain, usually related to skeletal involvement [1, 10]. In general, bone pain is described as a dull pain mainly localized to joints, legs and the back. The most severe expression of bone pain is bone crisis, reported in 30 to 65% of untreated patients and characterized by warmth and swelling of the affected site, often accompanied by systemic inflammatory signs [10–12]. Bone pain and particularly bone crisis are generally reported as well responsive to enzyme replacement therapy (ERT) [12].

However, we have recently observed pain recurrence or sensory dysesthesias with neuropathic features in a group of GD1 patients who had received long-term ERT (up to >20 years). Neuropathic pain is considered consequent to a primary lesion in somatosensory nervous system, in particular to the damage of small nerve fibers [13, 14]. However, small nerve fibers function has not been properly investigated in GD1.

## Methods

### Patients

This is a cross-sectional study that enrolled 25 GD1 patients (13 females, 12 males, aged 18 to 63 years) followed for 10 to >20 years by the Regional Coordinator Centre for Rare Diseases of the Academic Medical Centre Hospital (AMCH) of Udine. Twenty-nine GD1 patients who came to the annual follow-up visit from 1st April 2016 to 31st December 2016 were asked to participate in the study, regardless of the presence of painful symptoms. Twenty-five patients accepted to participate and signed the informed consent. The study was approved by the Ethical Committee of the Regione Friuli Venezia Giulia.

The diagnosis of Gaucher disease was based on the assessment of residual leukocytes β-glucosidase activity and the molecular analysis of the *GBA* gene. Twenty-two patients received alglucerase/imiglucerase therapy or substrate reduction therapy (SRT) with miglustat (1 patient) for a mean of 15.0 (±6.4) years, while 3 patients were naïve for any treatment.

The follow-up protocol included: yearly clinical examination, laboratory tests (Hb, platelets, leucocytes, ferritin, AST, ALT, GGT, ALP, direct and conjugated bilirubin, serum protein, gammaglobulins, lipid pattern) and bi-annual imaging study (liver and spleen volume measurement by echography and nuclear magnetic resonance, NMR; NMR bone marrow analysis; L1-L4 bone mineral density, BMD, by DEXA). Therapy efficacy was evaluated by Zimran-severity score index (Zimran-SSI).

Other causes of somatic and/or autonomic neuropathy were excluded: diabetes mellitus; renal, liver or thyroid dysfunction; HIV, HCV; hematological diseases; connective tissue diseases; malignancies; vitamin B12 deficiency; hereditary neuropathy; exposure to neurotoxic drugs; alcohol abuse; causes of monoclonal gammopathy not GD related.

### Bone pain

Anamnestic data on the presence of recurrent or chronic dull pain, (not movement related, localized to joint, legs or vertebral column) as well as on the presence of bone crisis were collected through patients’ diary consultation and direct interrogation. In presence of a positive feedback, pain was classified as of bone origin.

### Neuroalgologic evaluations

Neurological examination included extensive sensory evaluation according to the European Federation Neurological Society (EFNS) Guideline [15, 16]. Intensity of ongoing or shooting spontaneous pain, allodynia (static mechanical [pressure], dynamic mechanical [brush], heat or cold [thermal]), and hyperalgesia were graded with the 11-point numerical rate scale (NRS). The Douleur Neuropathique en 4 questionnaire (DN4) was preliminary assessed [17], if the DN4 score was at least 1/10 the Italian version of Neuropathic Pain Symptom Inventory (NPSI) was performed [18]. Presence and distribution (e.g. length or non-length dependent) of sensory loss and pain were recorded.

#### Nerve conduction studies tests

Sensory and motor nerve conduction studies (NCS) were performed using surface recording electrodes and standard placement. Compound muscle action potential (CMAP), motor nerve conduction velocity (MCV), distal motor latency (DL) and F wave latencies were recorded for median, ulnar, peroneal, and tibial nerves. Sensory nerve conduction velocity (SNCV) and sensory nerve action potential (SNAP) were assessed for median, ulnar, lateral femoral-cutaneous and sural nerves.

#### Quantitative sensory testing

QST was performed using calibrated device and standard procedure [19, 20]. Warm (WDT) and cold (CDT) thresholds as well as pain thresholds for cold (CPT) and hot (HPT) stimuli were assessed by the MedocTM device (MedocTM Thermal Sensory Analyser, TSA-2001, Israel), using a 30 × 30 mm probe with the method of limits. Abnormal sensations including paradoxical heat sensation (PHT) during alternating cold and warm stimulation, errata sensation, thermal allodynia or hyperalgesia, and aftersensation were recorded.

The mechanical detection threshold (MDT) was measured with a standardized set of modified von Frey hairs, from 0.26mN to 490mN using the method of limits making 5 threshold determination in the same sites of thermal thresholds determination. The VDT was performed with a graded Rydel-Seiffer tuning fork (64 Hz, 8/8 scale) placed over the head of homerous, scapula, ulnar styloid process and internal malleolus. The pressure pain threshold (PPT) was performed with a pressure gauge device (FDN200, Wagner Instruments, USA), able to exert forces up to 20 kg/cm2 with increasing ramp of 50 kPa/s. The PPT was determined with three series of ascending stimulus intensities, each applied as a slowly increasing ramp of 50 kPa/s. We created profiles of sensory changes using the Z-transformation of QST in order to obtain a Z-score [19, 20].

#### Skin innervation

Patients underwent a 3-mm punch skin biopsy at the distal site of the leg and proximal thigh following a standardized protocol [21]. Specimens were fixed (2% paraformaldehyde–lysine–sodium periodate, 4 °C overnight), cryoprotected, serially cut with a cryostat and immunostained using polyclonal anti-protein gene product 9.5 (Ultraclone Ltd). Intra-epidermal nerve fiber (IENF) density was calculated by two observers blinded to the diagnosis on three non-consecutive central sections by bright-field microscopy and compared to sex and age-adjusted normative values [22].

### Statistical analysis

Shapiro-Wilks statistic was used to test the normal distribution of variables. Clinical data, neurophysiologic measures and skin biopsy findings were compared by using the χ2 or Fischer exact tests, and the Mann-Whitney U-Test for comparisons of means. Data were reported as means and standard deviation (SD) when normally distributed, and median with 25 to 75% quartiles (interquartile range, IQR) when not normally distributed. *P* values <0.05 were considered statistically significant. IENF density was considered abnormal when it was below the 5th percentile of the normative database [22]. Findings for distal leg were compared with international normative data for distal leg age stratified [22], whereas those from the proximal thigh were compared with archive normative data [23] and with 86 age and sex-matched healthy subjects from our Laboratory.

## Results

Patients’ demographic characteristics and a summary of therapeutic outcomes are reported in Table 1. At therapy beginning (T0), patients’ mean age was 27.2 (±14.6) years with seven patients starting therapy during pediatric age (≤16 years). Twenty-one out of 25 patients received exclusively ERT, one received a ERT/SRT combination (patient 20) and three were naïve patients (patients: 2, 16, 24). The mean ERT period was 15.0 years (±6.4) with a mean initial dosage of 35.3 (±14.2) IU/kg/b.w., increased to 66.5 (±22.5) IU/kg/b.w. at last visit (Tx).

**Table 1:** Patients’ demographic and clinical data

Characteristic			*N* = 25		
Female n° (%)			12 (48)		
Male n° (%)			13 (52)		
Age at start ERT (years)					
Mean ± SD			27.2 ± 14.6		
Range			3–60		
Duration of treatment (years)					
Mean ± SD			15.0 ± 6.4		
Range			0–22		
Patients ≤16y old at start of treatment, n° (%)			7 (31.8)		
ERT variations and therapeutic results					
	Baseline - T0		Follow-up evaluation - Tx		
	Mean ± SD	(Range)	Mean ± SD	(Range)	P
ERT dose (U/Kg/Month)	35.3 ± 14.2	(15–60)	66.5 ± 22.5	(35–115)	-
Hemoglobin (g/dl)	11.9 ± 1.5	(9.4–14.8)	14.3 ± 1.6	(11.8–17.4)	<0.01
Platelets (×10.000/mm^3^)	155.7 ± 101.3	(42–379)	214.3 ± 94.4	(107–526)	<0.05
Gammaglobulin (mg/dL)	1632.5 ± 877.1	(587–4632)	1164.5 ± 339.6	(570–1832)	<0.01
Liver volume (MN)*	1.99 ± 0.82	(0.8–3.6)	0.93 ± 0.20	(0.53–1.33)	<0.01
Spleen volume (MN)*	11.48 ± 6.22	(1.3–20.3)	2.97 ± 1.59	(0.92–5.62)	<0.01
BMD (Z-score)	−1 ± 1.40	(−3.36;0.8)	−0.26 ± 1.28	(−2.9;1.7)	-
Pain	13		13		
Bone crisis	7		-		
Zimran severity score index**					
Mild (≥0 to 10)	16		21		<0.05
Moderate (≥11 to 25)	5		1		
Severe (>25)	1		-		

*MN: multiple of normal value

**Zimran Z-score was available for 22 patients

At T0, β-glucosidase activity was significantly reduced in all patients (data not shown), while the molecular analysis of the *GBA* gene showed the presence of the common N370S mutation in 21 patients (four homozygous and 17 compound heterozygous) while different mutations were identified in four patients, Table 2. In course of ERT we observed a significant reduction of liver and spleen volume (*p* < 0.01) and a progressive normalization of laboratory parameters: mean Hb value increased from 11.9 to 14.3 g/dl (p = <0.01) while platelets count increased from 155.7 × 10^3^/mm^3^ to 214.3 × 10^3^/mm^3^ (p = <0.05). Elevated serum levels of IgG were present in ten patients (patients: 5, 6, 7, 8, 10, 15, 16, 17, 21, 25) at T0 and persisted in two of them (patients 7 and 8) at Tx, with a mean value decreasing from 1632.5 to 1164.5 g/dl (p = <0.01).

**Table 2:** Patients’ molecular data and pain history

Patient No.	Sex M/F	Age Tx (yrs)	ERT duration (yrs)	Genotype		Bone Pain		Neuropathy pain localisation	NRS 0–10	Pain quality	DN4 score (0–10)
				Allele 1	Allele 2	T0	Tx	Tx	Tx	Tx	Tx
1	F	35	20	N370S	L444P	N	N	Lower limb	5	Cold	4
2	M	18	0	N370S	Rec NciI	N	---	-	0	-	0
3	F	40	17	N370S	D399N	N	N	-	0	-	0
4	M	18	15	N370S	D409H	N	N	-	0	-	0
5	M	54	19	R48W	L444P	J, BC	N	Feet	5	Cooling	4
6	F	53	19	R48W	L444P	J	N	-	0	-	0
7	F	35	21	R170P	c.1225-10delC; c.1225-14 T > A	J, BC, PF	H, UL	Toes, feet	3.5	Pinprick	4
8	M	37	21	R170P	c.1225-10delC; c.1225-14 T > A	J, BC, PF	J	Lower limbs	3.5	Tingling	4
9	F	32	22	N370S	L444P	J	N	-	0	-	0
10	M	51	17	N370S	Rec NciI	J, B, BC	N	-	0	-	0
11	F	42	17	N370S	g.4179_5042conJ03060.1: g.2367_2911	J, B, UL	N	-	0	-	0
12	M	42	17	N370S	g.4179_5042conJ03060.1: g.2367_2911	N	N	Lower limbs	6	Tingling	4
13	M	63	11	N370S	G202R	B, UL, LL	B	Lower limbs and back	6	Cooling	4
14	M	27	14	N370S	H255Q + D409H	N	N	-	0	-	
15	F	50	14	N370S	V214X	J, BC	N	Distal leg	7	Cooling	4
16	F	38	0	N370S	H255Q + D409H	N	---	Distal leg	5	Cooling	5
17	F	53	17	N370S	N370S	J, UL, LL	N	Lower limbs and back	8	Burning	5
18	F	44	13	N370S	V214X	N	N	Distal leg	8	Burning	6
19	M	20	13	N370S	N370S	N	N	-	0	-	2
20*	M	51	11	N370S	W381C	J	N	Distal leg	5	Cold pain	4
21	F	48	18	N370S	Rec NciI	N	N	Distal leg	5	Cold/Burning	5
22	M	46	19	N370S	g. − 3091 + 834del3925	J, BC	J, B	-	0	-	0
23	F	48	20	N370S	L444P	J, B, BC	N	-	0	-	0
24	F	54	0	N370S	L444P	N	---	Lower limbs	5	Tingling/Burning	4
25	M	35	20	N370S	N370S	N	N	-	0	-	0

* Patient receiving SRT and ERT

Genotype: Missense and nonsense mutations are reported using the traditional protein mutation nomenclature for GBA which considers position 1 the first aminoacid of the processed protein which lacks 39 aminoacids of the leader sequence (reference sequence AAC63056.1). These mutations are presented without “p.” in the mutation name. Intronic mutations are described as recommended, considering nucleotide +1 the A of the first ATG translation initiation codon. Nucleotide numbers are derived from the GBA1 cDNA (GenBank reference sequence NM_000157.1). In the case of the deleted allele, the genomic nucleotide position is used, according to the GBA (GenBank accession no. J03059.1) and pseudo-GBA (GenBank accesion no. J03060.1) sequences

Pain findings: B: back; BC: bone crisis; H: hands; J: joints; LL: lower limbs; N: no; PF: pathological fractures; UL: upper limbs

Bone mineral density showed a mean Z-score of −1.0 at T0 that increased to −0.26 at last visit. Only three patients (patients: 7, 8, 20) had a Z-score in the range of osteoporosis (−2.0, −2.3 and −3.4 respectively) that after ERT normalized in patients 7 and remained pathological in the other 2 (8 and 20): −2.0 and -2.8 respectively.

Zimran-SSI was calculated in 21 patients both a T0 and Tx, showing a reduction to the milder severity group (score 0–10) in all of them.

Due to the severe osteoporosis, patient 20 received exclusive SRT with miglustat for 3 years, then he switched to combined SRT/ERT for 1 year and finally switched to ERT exclusively. He never complained of bone crisis or symptoms secondary to miglustat treatment (diahrrea, abdominal pain, tremors).

Data of patients’ therapy duration, genotyping and pain history are reported in Table 2. At T0 a history of bone pain was reported in 13 of the 22 treated patients, 59.1%, (patients: 5, 6, 7, 8, 9, 10, 11, 13, 15, 17, 20, 22, 23), including two siblings (patients 7 and 8) who suffered also from pathological fractures before ERT start. Seven patients, 31.8%, presented recurrent bone crisis episodes (patients: 5, 7, 8, 10, 15, 22, 23). In course of ERT both bone crises and severe joint pain disappeared in all patients, while attenuated painful symptoms involving joints, legs and the back remained still present in 4 patients, 18.1% (patients: 7, 8, 13, 22).

However, at Tx a group of 13 patients (patients: 1, 5, 7, 8, 12, 13, 15, 16, 17, 18, 20, 21, 24) referred the presence of painful symptoms, described as a persistent *cold pain* with proximal distribution, in six with paroxysmal cold sensation. Among them: eight had referred bone pain at T0 (patients: 5, 7, 8, 13, 15, 17, 20, 21), three developed pain over time (patients: 1, 12, 18) and two were new diagnosed patients, naïve to any treatment (patients 16 and 24). At NRS, pain intensity resulted >4/10 in all. Only one patient (17) experienced a severe chronic pain (mean NRS of 8) with pins paroxysmal (NRS of 10), Table 3. All patients described this cold painful sensation as completely different from the pain at T0. Aching sensation in the limbs and joints was also reported by patients 7, 8, 22.

**Table 3:** Neuroalgological, functional and hystopathological data at Tx

Patient No.	Sex M/F	ERT duration (yrs)	NPSI* NRS (0–10)				QST at dorsal foot Z score					IENF/mm (lower limit of normality)	
			Q1	Q10	Q11	Q12	WDT	CDT	PHS	MDT	PPT	DL	Pth
1	F	20	5	5	0	0	+2.6	−3.9	+3.8	−2.5	−0.1	6.2 (7.1)	3.2 (14.3)
2*	M	0	−	−	−	−	+2.1	−3.3	+2.9	−3.1	−0.3	−	−
3	F	17	−	−	−	−	+2.3	−3.5	+2.8	−2.8	−0.1	3.8 (5.7)	6.6 (14.0)
4	M	15	−	−	−	−	+2.4	−3.1	+3.1	−3.4	−0.3	6.8 (10.9)	3.1(16.4)
5	M	19	7	6	0	5	+1.9	−3.3	+3.3	−2.5	−0.2	0.8 (3.5)	6.2 (13.5)
6	F	19	−	−	−	−	+1.6	−3.3	+2.9	−2.6	+2.8	−	−
7	F	21	0	7	6	0	+2.1	−2.8	+2.8	−2.9	−0.2	−	−
8	M	21	0	2	0	7	+2.3	−2.6	+2.8	−3.1	+3.1	8.5 (5.2)	7.8 (14.1)
9	F	22	−	−	−	−	+2.4	−3.0	+2.2	−2.8	−0.4	0.6 (7.1)	0.9 (14.3)
10	M	17	−	−	−	−	−	−	−	−	−	4.4 (3.5)	6.5 (13.5)
11	F	17	−	−	−	−	−	−	−	−	−	4.4 (5.7)	7.2 (14.0)
12	M	17	5.5	5	0	0	+2.6	−2.7	+2.4	−2.5	+3.1	5.2 (4.4)	6.2 (13.8)
13	M	11	6	5	3	6	−	−	−	−	−	8.2 (2.8)	20.0 (11.2)
14	M	14					+2.7	−3.3	+2.7	−2.4	−0.3	−	−
15	F	14	7	2	4	0	−	−	−	−	−	8.2 (4.3)	7.3 (13.8)
16*	F	0	8	5	7	0	+2.8	−3.8	+2.6	−2.6	+3.4	2.8 (7.1)	4.6 (14.3)
17	F	17	10	4	6	0	+2.6	−3.1	+3.1	−2.8	+2.8	9.8 (4.3)	9.6 (13.8)
18	F	13	8	6	7	5	+2.7	−3.9	+3.0	−2.7	+3.2	7.2 (5.7)	13.0 (14.0)
19	M	13	0	0	0	0	−	−	−	−	−	3.8 (6.1)	17.5 (16.4)
20**	M	11	6	4	0	4	+2.8	−3.2	+2.1	−3.1	+0.2	6.2 (3.5)	9.8 (13.5)
21	F	18	5	1	0	7	−	−	−	−	−	6.0 (5.7)	9.9 (14.0)
22	M	19	−	−	−	−	−	−	−	−	−	3.7 (5.7)	7.6 (13.8)
23	F	20	−	−	−	−	+2.6	−3.1	+2.9	−3.2	−0.1	7.5 (5.7)	5.2 (14.0)
24*	F	0	5	3	0	0	+2.5	−3.6	+2.7	−3.0	+0.3	1.0 (4.3)	2.5 (13.8)
25	M	20	−	−	−	−	−	−	−	−	−	5.2 (5.2)	6.2 (14.3)

*Patients naïve to any therapy; ** Patient receiving SRT and ERT

Neuroalgological findings. *NPSI: Q1, Q10, Q11, Q12 choose by relevance. Q1 examines spontaneous cooling/burning pain, Q10 pain evoked by cold, Q11 spontaneous pins pain and Q12 pareaesthesia

Sensory thresholds (WDT, CDT, PHS, MDT) at QST refer to the dorsal foot and are reported as Z score. Values ±2.5 was considered abnormal

IENF density (IENF/mm) was considered abnormal when it was below the 5th percentile of the normative database. Finding for distal leg were compared with international normative data for distal leg age stratified and for proximal sites with published normative data not age stratified and also 86 age and sex-matched healthy subjects from our Laboratory (not published). IENF densities at proximal thigh were the following: for females 13.5 ± 2.3 (range 9.8–16.3); males 12.8 ± 2.7 (range 8.6–16.4). In detail, the mean normal values age and sex-stratified for proximal thigh were for females: <19 yrs.: 16.3; 20-29 yrs.: 15.0; 30-39 yrs.: 14.3; 40-49 yrs.:14.0; 50-59 yrs.: 13.8; 60-69 yrs.: 11.8; 70-79 yrs.: 10.2; 80-89 yrs. 9.8. For males: <19 yrs.: 16.4; 20-29 yrs.: 15.2; 30-39 yrs.: 14.1; 40-49 yrs.:13.8; 50-59 yrs.: 13.5; 60-69 yrs.: 11.2; 70-79 yrs.: 9.8; 80-89 yrs.: 8.6

**Fig. 1:**
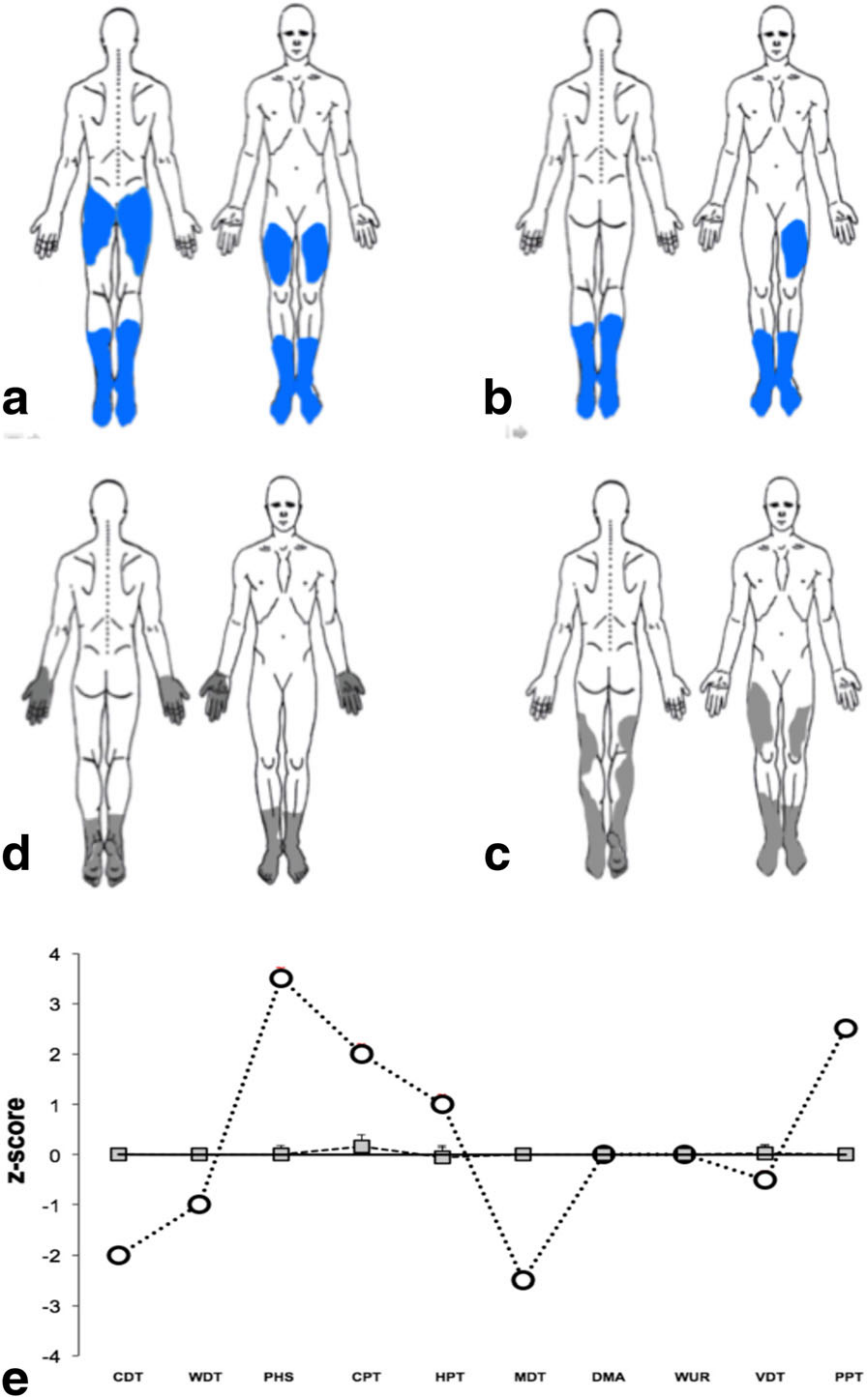
Detail drawing of painful symptoms (**a-b**) and negative sensory signs (**c**-**d**). **a** and **b**: the painful area (*ongoing* cold pain) of two representative GD patients (paitents 5 and 8, respectively). **c-d**: the pinprick hypoalgesia in the same patients. **e** representative example of the sensory profile at QST at the proximal thigh (patient no. 12) (Z-score). Z-values above “0” indicate a gain, z-value below “0” a loss of sensory function, whereas z-values > ±2 rated pathological. Paradoxycal Heat Sensation (PHS) is here considered as a sign of gain of function. The other abbreviations are detailed in the text

Clinical evaluation showed pinprick hypoesthesia at lower limb, usually in patchy fashion, in nine patients (patients: 8, 9, 12, 15, 16, 17, 18, 20, 21), in two of them (patients 8 and 18) with a stocking pattern involving also the hands, Fig. 1. In four patients (patients: 8, 9, 13, 17) distal tactile hypoesthesia was found while ankle reflexes were reduced in three patients (patients: 12, 13, 20). Neither static or dynamic mechanical allodynia nor hypopallesthesia were observed.

In all 13 patients with chronic pain at Tx, the DN4 score was ≥4/10, Table 2. The data of NPSI questionnaire (Table 3) showed spontaneous cooling-pain (Q1) in a group of 11 patients (NRS > 4; mean NRS 6.1 ± 1.6) and evoked pain (Q10) in another group of 11 patients (NRS > 4; mean NRS 7.2 ± 1.1). Cold evoked burning pain (Q10) was present in 9 (mean NRS 4.2 ± 1.8), pain evoked by pressure (Q9) in 2 (patients 15 and 21; mean NRS 4.5; data not shown in Table 3) whereas two groups of patients, composed by six patients each (Q11 and Q12), showed pins evoked sensation (mean NRS 5.5 ± 1.6) and tingling paraesthesia (mean NRS 5.6 ± 1.2), Table 3.

Seventeen patients performed the multimodal QST, Fig. 1. The sensory profile showed a stereotypical pattern with high cold thresholds (CDT) with *errata sensation* (burning or pins instead of cold), presence of paradoxycal heat sensation (PHS) and mechanical hypoestesia (MDT), Table 3. Finally, six patients presented pressure pain hyperalgesia (PPT), Table 3. Sensory and motor nerve conduction studies were normal in 22 patients. One patient (13) had carpal tunnel syndrome and 2 patients (patients 5 and 13) showed L5-S1 radiculopathy not related to GD.

**Fig. 2:**
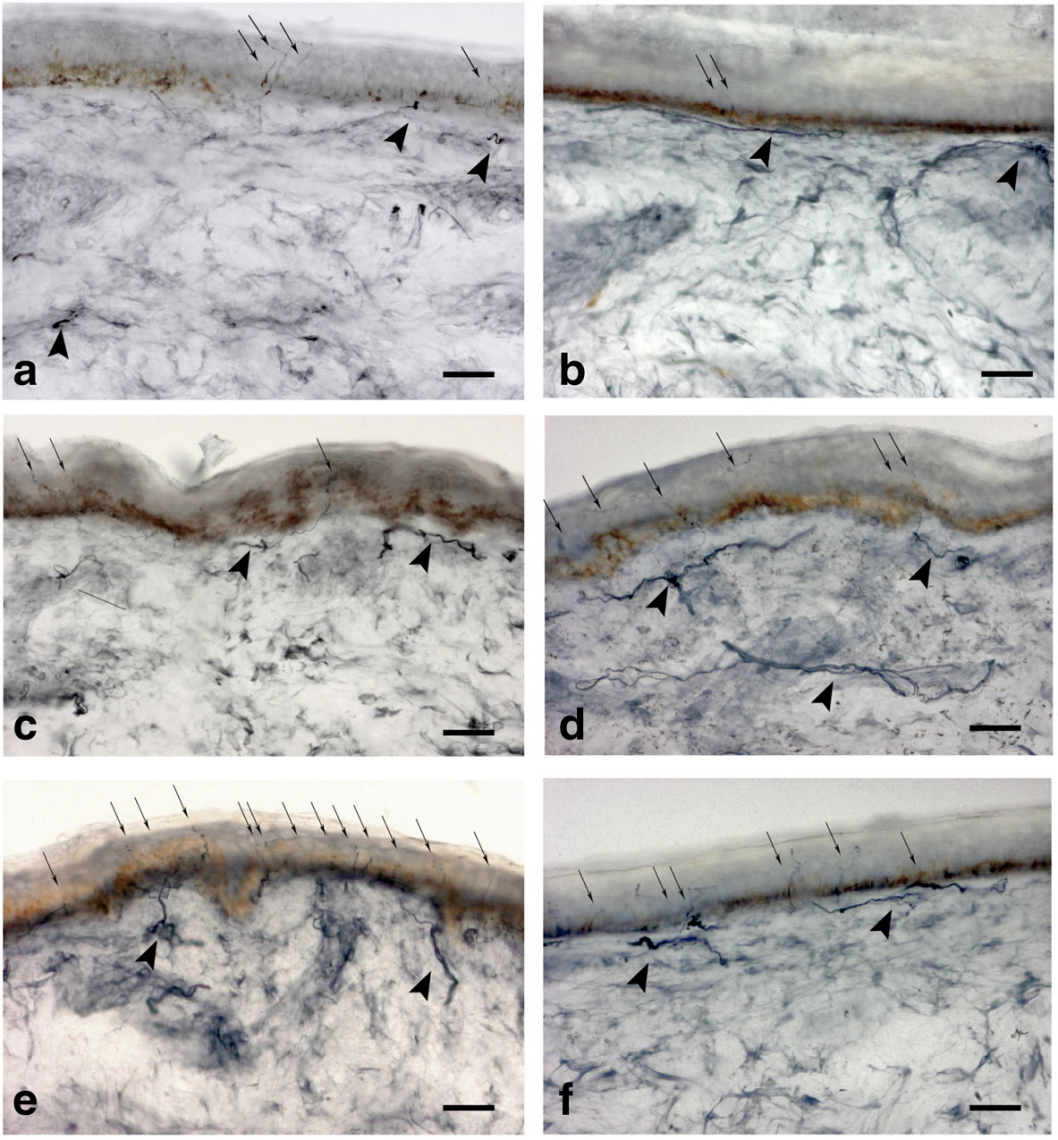
Skin biopsy samples at proximal thigh (**a-c-e**) and distal leg (**b-d-f**) from a GD1 patients with dying-back skin denervation (**a-b**), with non lenght-dependent pattern (**c-d**), and in healthy subject (**e-f**). Arrows indicate intraepidermal nerve fibers, arrowheads indicate dermal nerve bundles. The density of intraepidermal nerve fibers is reduced in A-B-C. Bright-field immunohistochemistry in 50 μm sections stained with polyclonal rabbit anti- protein-gene-product 9.5 antibody. Bar = 60 μm

Skin biopsy performed in 21 patients, showed abnormal IENF density in 19 of them (patients: 1, 3, 4, 5, 8, 9, 10, 11, 12, 15, 16, 17, 19, 20, 21, 22, 23, 24, 25), Table 3. Seven patients presented a length-dependent loss of fibers (patients: 3, 5, 9, 16, 19, 22, 24), whereas 12 patients (patients: 1, 4, 8, 10, 11, 12, 15, 17, 20, 21, 23, 25) showed a not length-dependent pattern of epidermal denervation, Fig. 2. Skin denervation was found in nine patients only at proximal site, Table 3. We found a significant inverse correlation between IENF density and CDT at both the distal and proximal site (Pearson coefficient = 0.7 at distal leg and 0.77 at proximal site). No correlation between pain intensity and IENF density or *GBA* genotype was observed.

## Discussion

Despite affecting a considerable number of patients and representing one of the most disabling symptoms, pain per se has been poorly studied in GD. It has been generally associated with skeletal involvement, particularly with its most severe form, the bone crisis [1, 24]. Moreover, this association has been reinforced by the positive effect of ERT on bone symptoms. However, patients seldom refer also abdominal pain or a discomfort sensation due to hepatosplenomegaly and only recently pain has been related to peripheral neuropathy [5–7, 9, 25, 26].

Muscoskeletal pain is defined by the presence of acute or chronic pain that arises from actual or threatened damage to *non-neural tissue*, and is due to the activation of nociceptors by several noxius stimuli such as chemical mediators release or mechanical stress [27]. The skeletal pain in GD patients is generally a localized joint pain often referred as a chronic deep penetrating pain and tenderness sensation. However, some patients may experience severe and dramatic acute pain (bone crisis) usually related to ischemic insult to the bone tissue [10].

The persistence of pain symptoms in several patients after long-term ERT, even after they had reached the therapeutic goals [28], with modified sensation (i.e. widespread burning-cold, pins sensation on leg and back regions) and negative signs as hypoesthesia or hypoalgesia, prompted us to investigate its possible neuropathic origin. In the past decade the presence of sensory symptoms in GD1 has been reported in different studies in almost 30% of patients: cold sensation (13%–28.4%), pins and needle (15%–25%) and on-going burning sensation (11%–33%) [6, 7, 9, 29, 30]. However, a systematic evaluation of small sensory nerves has not been performed. Only one previous study analyzed small nerve function using an obsolete and not adequate system: the Current Perception Threshold test [5]. This study for the first time provides a comprehensive analysis of the pain symptomatology in a cohort of 25 GD1 patients.

In our cohort ERT showed to be effective in controlling bone involvement and bone pain in nine out of the 13 who presented bone pain at T0. Only a group of four patients showed a persistence of attenuated joint pain during treatment (patients: 7, 8, 13, 22). However, an evolution of painful symptoms was observed after long-time ERT, with the presence of pain features suggestive of neuropathic origin with a quite stereotypical pattern of sensory profile in 13 patients. Four of them belonged to the group in which bone pain was resolved in course of ERT (patients: 5, 15, 17. 20,), 3 were patients with persisting bone symptoms (patients: 7, 8, 13), 4 were previously asymptomatic (patients: 1, 12, 18, 21) and 2 were new diagnosed (patients: 16 and 24). The pattern mostly showed thermal-hypoesthesia affecting lower limbs with prevalent involvement of cold than warm perception, similar to pain features observed in Fabry patients [31, 32].

These functional data were confirmed by histopathology, showing the presence of IENF denervation in 19/21 patients, both symptomatic and asymptomatic ones. Interestingly a denervation pattern was observed also in the three naïve asymptomatic patients (patients: 2, 16 and 24), excluding a correlation with ERT.

The presence of SFN in asymptomatic patients, strongly suggests a *constitutive* role of peripheral neuropathy in GD. Most interesting, we found a high prevalence of a non-length dependent degeneration of somatic IENF, suggesting the primary involvement of dorsal root ganglion (DRG) neurons.

The presence of a severe SFN is a well-known feature in Fabry disease and has recently been described also in a patient affected by Pompe disease [33–35]. Despite the hystopathological evidences, the molecular mechanisms underlying painful perception in patients affected by these lysosomal diseases are still poorly understood. However, evidences of a direct link between substrate accumulation and pain have been provided. Indeed, the administration of lyso-Gb3 to healthy mice caused mechanical allodynia and evoked an increase in intracellular Ca^2+^ levels associated with the functional up-regulation of voltage-activated Ca^2+^ channels in DRG neurons [36] These data suggest that the lipids species that accumulate in Fabry disease may cause pain through a direct action on sensory neurons, being a Ca^2+^-dependent excitability of nociceptors a possible mechanism. In addition, a direct effect of lipid accumulation on small nerve fibers damage has been hypothesized, since Ca^2+^ influx promoted by lyso-Gb3 may cause Ca^2+^-dependent excitotoxicity [37].

Whether a similar mechanism plays a role in painful perception in Gaucher patients needs to be investigated. However, it has been well documented that increased levels of glucosyceramide, the main lipid accumulating in GD, leads to an overactivation of the endoplasmic reticulum (ER) calcium channel, the ryanodine receptor, with the consequent increase of agonist-inducted Ca2+ release from this compartment [38, 39]. Furthermore, it has been suggested that the Ca^2+^ release from ryanodine-sensitive intracellular stores can induce neuronal cell death [38]. In light of these data it is reasonable to hypothesize a direct link between lipid storage and pain also in GD. Further experiments are needed to confirm this hypothesis.

## Conclusions

The results of our study suggest that SNF is a *constitutive* feature of the disease in GD. This result stresses the concept of the existence of a phenotypic continuum already evidenced by previous studies [3]. Therefore, pain might be considered as a “late-onset” complication of the disease that can manifest even after long-term ERT as a consequence of a structural damage of peripheral nervous system. These results suggest the need to differentiate between bone and neurological pain in GD1 in order to provide an appropriate anti-pain therapy and to avoid unnecessary ERT dose escalation with the consequent unjustified increase of health costs. Future follow-up studies might also help to understand the natural progression of SNF in Gaucher disease.

## Abbreviations

CDT: Cold threshold
CMAP: Compound muscle action potential
CPT: Cold pain threshold
DL: Distal motor latency
DN4: Douleur neuropathique en 4 questionnaire
ERT: Enzyme replacement therapy
GD: Gaucher disease
HPT: Hot pain threshold
IENF: Intra-epidermal nerve fiber
MCV: Motor nerve conduction velocity
MDT: Mechanical detection threshold
NCS: Nerve conduction studies
NPSI: Neuropathic Pain Symptom Inventory
NRS: 11-point numerical rate scale
PHT: Paradoxical heat sensation
QST: Quantitative Sensory Testing
SNCV: Sensory nerve conduction velocity
SNAP: Sensory nerve action potential
SNF: Small nerve fibers
SRT: Substrate reduction therapy
VDT: Vibratory detection threshold
WDT: Warm threshold
Zimran-SSI: Zimran-severity score index
